# Quantitative Assessment of Left and Right Atrial Strains Using Cardiovascular Magnetic Resonance Based Tissue Tracking

**DOI:** 10.3389/fcvm.2021.690240

**Published:** 2021-06-24

**Authors:** Yang-Yang Qu, Dominik Buckert, Gen-Shan Ma, Volker Rasche

**Affiliations:** ^1^Internal Medicine II, Ulm University Medical Center, Ulm, Germany; ^2^Department of Cardiology, Zhongda Hospital, School of Medicine, Southeast University, Nanjing, China

**Keywords:** cardiovascular magnetic resonance, tissue tracking, left atrial, right atrial, strain, normal value

## Abstract

**Background:** Left and right atrium (LA and RA) exert an essential and dynamic role in ventricular filling and hence affect heart performance. Strain quantification has been reported as a novel parameter to assess function. However, the assessment of bi-atrial strains with cardiovascular magnetic resonance (CMR) based techniques is still limited and gender- and age-specific normal values in a healthy population are missing.

**Methods:** One hundred and fifty healthy volunteers (49.8 ± 17.3 years, 75 males) undergoing 1.5 Tesla CMR examination were retrospectively and consecutively recruited. LA and RA free wall (RAFW) radial and longitudinal strains (RS and LS) associated with atrial reservoir, conduit and booster pump functions were evaluated with CMR based tissue tracking (CMR-TT) technique.

**Results:** The reservoir, conduit and pump LS resulted as 30.7 ± 10.2%, 19.5 ± 8.2%, 10.9 ± 3.7% for LA, and 52.2 ± 17.6%, 33.3 ± 14.2%, 19.1 ± 8.5% for RAFW, respectively. The amplitude of RA strains was significantly larger than that of LA strains, except for conduit RS. With the increase of age, the decrement of majority of reservoir and conduit strains were observed, while pump strains remained unaffected. Females presented with significantly larger RAFW strains compared with males, especially in the elderly. In addition to the positive correlation between atrial strains and emptying fraction, the negative correlation between atrial strains and volume index was also confirmed. Intra-observer reproducibility of LA strains was superior to RAFW strains (coefficient of variation: 10.12–17.04% vs. 10.80–27.36%, respectively), and the measurement of reservoir and conduit strains was more reproducible in comparison with pump strain.

**Conclusion:** CMR-TT is a feasible and reproducible technique to quantify LA and RA strains and determine atrial phasic functions. The existence of age- and gender-related difference of strains suggests the necessity to establish specific normal values for individual populations.

## Introduction

The left and right atrium (LA and RA) play an essential and phasic role in modulating ventricular filling through reservoir, conduit and booster pump functions to maintain the normal cardiac hemodynamics (1–4). Conventionally, atrial function is evaluated with diameter and volumetric analysis with echocardiography or cardiovascular magnetic resonance (CMR) (5). More recently, strain has been utilized to quantify atrial deformation and detect atrial dysfunction in various diseased conditions, such as hypertension (6), atrial fibrillation (7), myocardial infarction (8), non-ischemic cardiomyopathies (9, 10) and heart failure (11).

Similar to speckle tracking echocardiography (STE), CMR based tissue tracking (CMR-TT) is based on segmentation and tracking of myocardial tissue voxels throughout the cardiac cycle. It has been proven as a promising approach to assess left and right ventricular myocardial strains from routinely available steady-state free precession cine images (12–15). Due to the thin myocardial wall, insertion of appendage and vena cava, angle dependency and limitation of acoustic windows, the volumetric and mechanical analysis of atrial is challenging using STE (16). In contrast, CMR has emerged as the gold standard to evaluate the morphology and function of the atrium because of its high spatial resolution. The feasibility and reproducibility of CMR-TT in evaluating strain-based atrial function have been shown in prior studies (16, 17) and clear advantages over CMR tissue tagging and the possible inclusion into the clinical workflow have been reported (18, 19).

The establishment of normal values of atrial strains is fundamental to differentiate between normal and pathologic atrial deformation. Given that atrial function is associated with age and gender (20, 21), it is necessary to establish gender- and age-specific normal values of atrial strains for individual population. By now, quantitative assessment of bi-atrial strains, and here especially RA strains, with CMR-TT is limited. In this study, we aimed to comprehensively investigate the normal values of LA and RA reservoir, conduit and pump strains, including radial and longitudinal strains (RS and LS), in a relatively large healthy population. Gender- and age-related differences are investigated and related with baseline characteristics (including age, blood pressure, heart rate, ventricular ejection fraction, atrial volume index and atrial emptying fractions).

## Methods

### Study Participants

One hundred and fifty healthy adults (75 males and 75 females, 49.8 ± 17.3 years of age) referred for CMR examination were retrospectively and consecutively enrolled in this study. All participants had no signs of cardiovascular diseases (e.g., coronary artery disease, valvular heart disease, hypertension) or histories of the associated risk factors (e.g., smoking, diabetes, hyperlipidemia). The biventricular ejection fraction (EF) was proven normal [left ventricular EF (LVEF) ≥55%, right ventricular EF (RVEF) ≥45%] by routine echocardiography. Subjects with contraindications to standard CMR scanning were excluded as described before (12). All participants were subdivided into three age groups (G_20−40_, G_41−60_, and G_61−80_) with the same number of males and females in each group. The study was approved by the local ethics committee, and informed consents were obtained from all subjects.

### CMR Examination

The study participants underwent a standard CMR examination on a clinical 1.5 Tesla whole body scanner (Achieva, Philips Healthcare, Best, The Netherlands). All data were acquired with a dedicated cardiac 32-channel phased-array receive coil. The detailed scanning parameters have been described before (12). Briefly, CMR images covering the whole heart were acquired with an electrocardiogram-gated breath-hold steady-state free precession sequence. The bi-atrial phasic volumes were derived from the horizontal (4-chamber) and vertical (2-chamber) long axis views applying a commercially available post-processing software CVI^42^ (Circle Cardiovascular Imaging Inc., Calgary, Canada).

The maximal volume (V_max_) was obtained at end-systole phase of the cardiac cycle, right before the opening of the valves. Pre-atrial volume (V_preA_) was attained just before atrial contraction. The minimal volume (V_min_) was measured at late end-diastole at which atrial volume reached the lowest after atrial contraction and before valve closure (17). The phasic functions of atrium were assessed according to the following equations (9): (i) reservoir function: total emptying fraction (EF_t_) = (V_max_ – V_min_)/V_max_ × 100%; (ii) conduit function: passive emptying fraction (EF_p_) = (V_max_ – V_preA_)/V_max_ × 100%; (iii) booster pump function: active emptying fraction (EF_a_) = (V_pre−A_ – V_min_)/V_preA_ × 100%. V_max_ and V_min_ were normalized by body surface area prior to analysis.

### CMR-TT Analysis of Atrial Function

**Figure 1:**
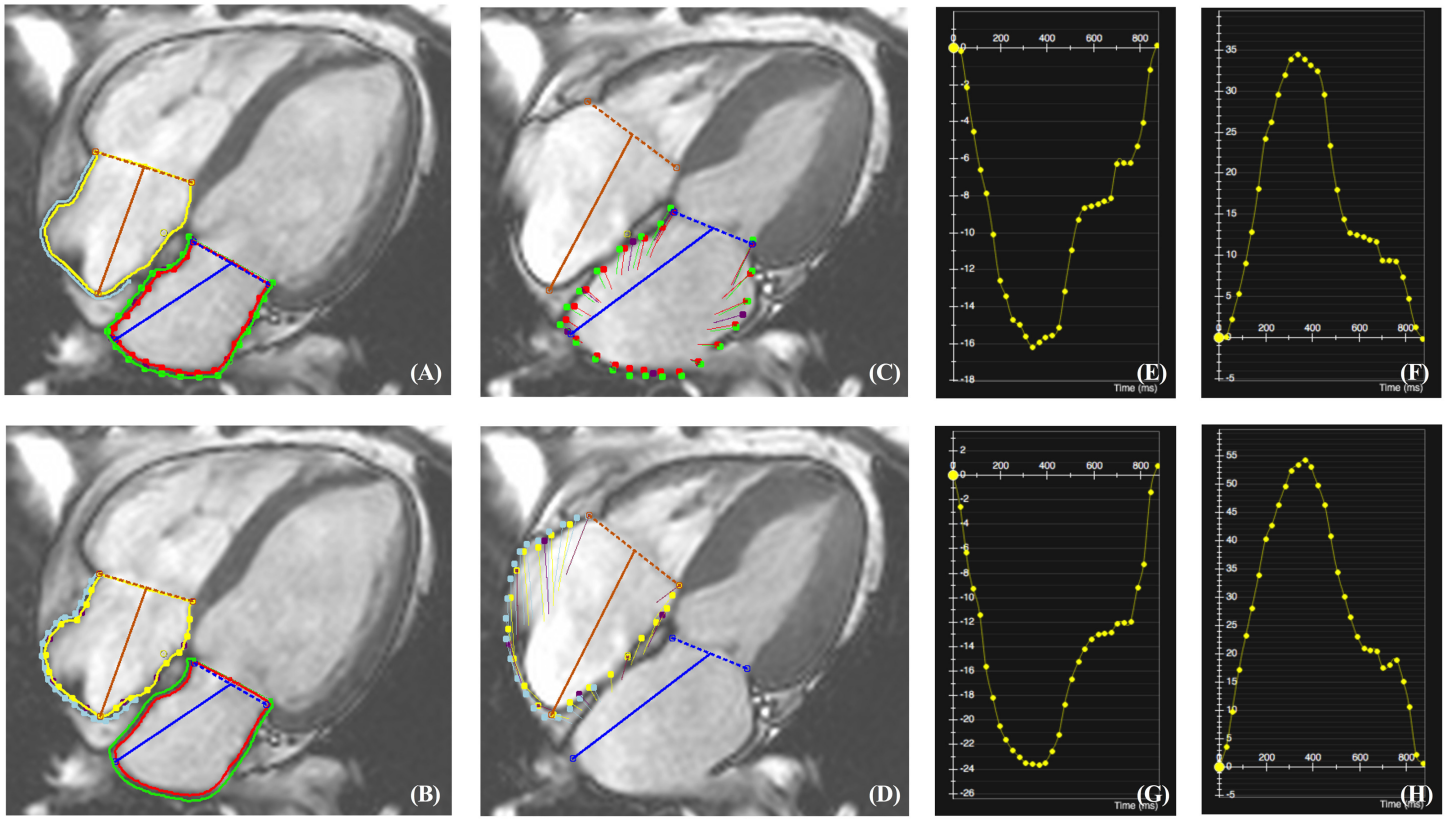
CMR-TT analysis of LA and RAFW radial and longitudinal strains (RS and LS). The location of mitral and tricuspid valves as well as the delineation of LA and RAFW endo- and epicardial borders were performed at end-diastole **(A,B)**. After the completion of tracking, the myocardial deformation at end-systole was indicated by red and green lines for LA **(C)**, and yellow and blue lines for RAFW **(D)**. The strain-time curves of LARS **(E)**, LALS **(F)**, RAFW-RS **(G)**, and RAFW-LS **(H)** were generated directly by CMR-TT.

**Figure 2:**
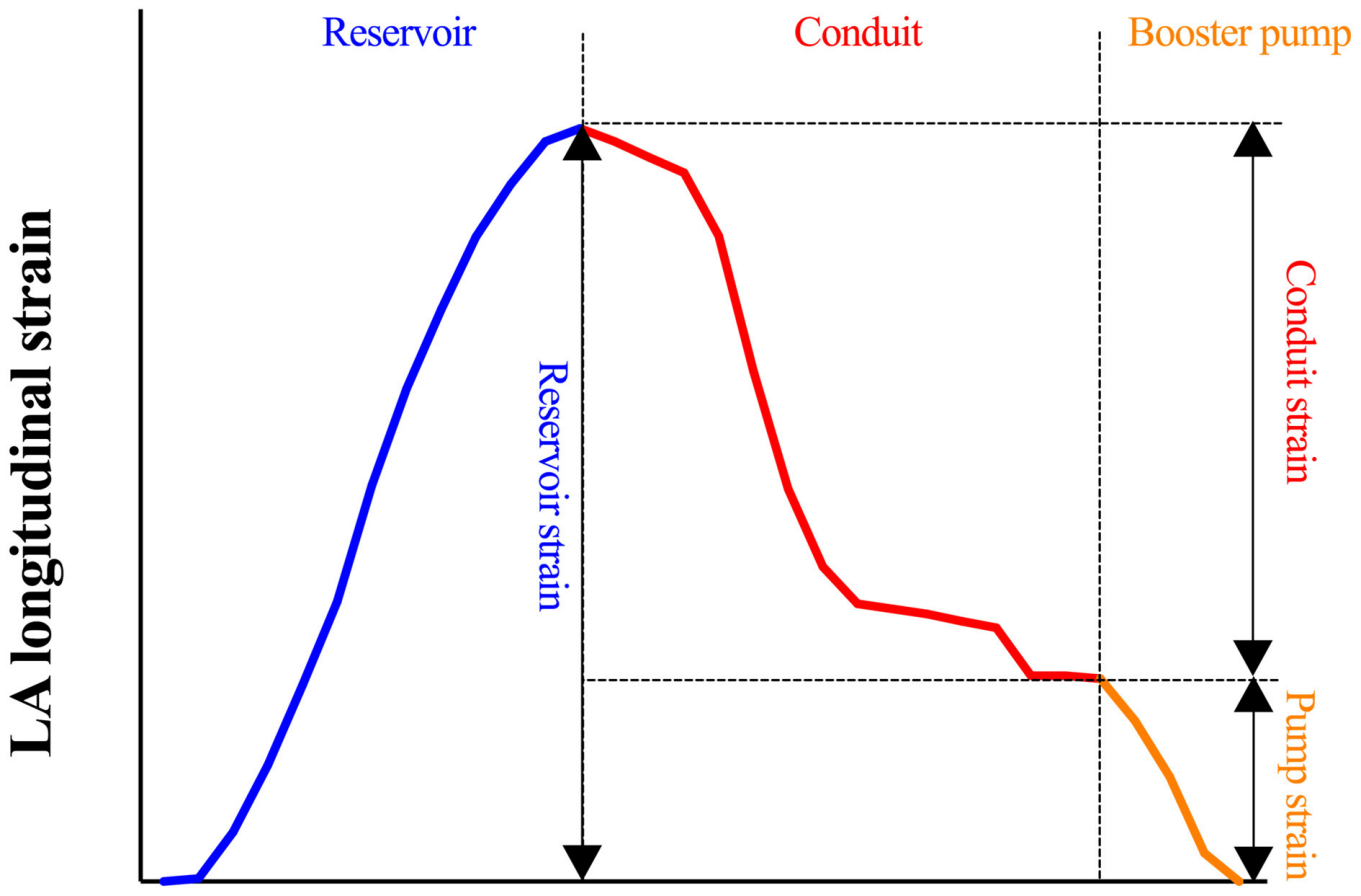
Representative plot illustrating the calculation of reservoir, conduit and pump LALS in Figure 1F.

Atrial function was quantified applying a commercially available post-processing software CVI^42^. It provides a rapid and simple procedure to manually locate mitral and tricuspid valves, semi-automatedly delineate LA and RA endo- and epicardial boundaries at end-diastole (Figures 1A,B) and present tracked boundaries at end-systole (Figures 1C,D). In order to diminish the influence of LA, RS, and LS of RA free wall (RAFW) rather than the whole RA myocardium were measured. Additionally, the confluence of the superior vena cava, pulmonary veins and the atrial appendage were excluded from the analysis. Subsequently, strain-time curves were generated to visually exhibit the dynamic strain of LA and RAFW (Figures 1E–H). Atrial reservoir, conduit and pump strains corresponding to atrial reservoir, conduit and booster pump functions were calculated (9) and recorded for the subsequent analysis (Figure 2).

In order to validate the reproducibility of CMR-TT technique in quantitatively assessing LA and RAFW strains, a second analysis was performed by the same observer in 20 randomly selected subjects 1 month after the initial analysis.

### Statistical Analysis

Continuous variables were expressed as mean ± standard deviation (SD). The independent sample *t*-test or non-parametric test was chosen to evaluate the differences of continuous variables between males and females according to the distribution of data. Paired sample *t*-test or Wilcoxon signed-ranks test was chosen to compare LA and RAFW corresponding strains, if appropriate. Comparisons among three age groups were completed using analysis of variance (ANOVA) or Kruskal-Wallis test followed by Bonferroni correction, as applicable. The Pearson or Spearman correlation coefficient (*r*) was evaluated to test the correlation between atrial strains and baseline variables. The intra-observer reproducibility was determined by intra-class correlation coefficient (ICC) and coefficient of variation (CoV). A two-sided *P* < 0.05 was considered statistically significant. All statistical analyses were performed with IBM SPSS Statistics, version 24.0 (IBM Corporation, Armonk, New York).

## Results

### Characteristics of the Study Participants

The baseline characteristics as well as morphological and functional parameters of atriums are summarized in Table 1. With the normal aging, the decrement of heart rate and increment of systolic heart pressure were observed (*P* < 0.05) with the absence of gender-related difference. In addition to the reduction of LA V_max_ and V_min_ index (VI_max_ and VI_min_), the decrease of LA EF_t_ and EF_p_ were noted among three age groups (*P* < 0.05). There was no gender-related difference of LAVI_max_ and LAVI_min_ (*P* = 0.90 and 0.38, respectively). In contrast, we found that RAVI_max_ and RAVI_min_ were significantly larger in males compared with females (*P* < 0.05). Unlike EF_t_ and EF_p_ decreasing with aging, EF_a_ of RA exhibited an increased tendency (*P* < 0.05).

**Table 1:** Baseline characteristics.

	**G**_****20−40****_		**G**_****41−60****_		**G**_****61−80****_		**Males**	**Females**	**All**
	**Males**	**Females**	**Males**	**Females**	**Males**	**Females**	**(*n* = 75)**	**(*n* = 75)**	**(*n* = 150)**
**Baseline demographics**									
Age (yrs)	29.5 ± 5.5	29.4 ± 7.4	50.8 ± 5.7	51.2 ± 6.5	69.3 ± 6.1	68.8 ± 5.7	49.9 ± 17.3^†^	49.8 ± 17.5^†^	49.8 ± 17.3^†^
HR (bpm)	74.0 ± 18.7	71.7 ± 11.5	66.3 ± 16.0	70.9 ± 14.6	63.8 ± 14.1	68.6 ± 11.2	68.0 ± 16.7^†^	70.4 ± 12.4	69.2 ± 14.7^†^
SBP (mmHg)	122.5 ± 13.1	116.2 ± 13.8	118.3 ± 11.3	122.7 ± 17.1	130.0 ± 14.7	126.7 ± 19.3	123.7 ± 13.8^†^	120.8 ± 21.6	121.5 ± 20.2^†^
DBP (mmHg)	67.8 ± 9.2	65.4 ± 8.9	70.1 ± 10.8	66.3 ± 8.3	69.2 ± 14.0	62.8 ± 9.1	69.0 ± 11.4	65.3 ± 8.5	66.7 ± 11.1
**LA and RA morphology and function**									
LAVI_max_ (mL/m^2^)	32.9 ± 8.4	32.3 ± 6.7	36.4 ± 11.9	37.4 ± 9.7	40.4 ± 13.7	38.5 ± 9.8	36.6 ± 11.8	36.1 ± 9.1^†^	36.3 ± 10.5^†^
LAVI_min_ (mL/m^2^)	11.0 ± 3.3	10.0 ± 3.1	14.5 ± 8.4	13.6 ± 5.1	17.8 ± 10.0	15.7 ± 6.5	14.5 ± 8.2^†^	13.1 ± 5.6^†^	13.8 ± 7.0^†^
RAVI_max_ (mL/m^2^)	38.3 ± 7.4	33.0 ± 6.6^*^	39.0 ± 11.1	34.4 ± 9.3	38.5 ± 12.0	31.9 ± 9.3	38.6 ± 10.2	33.1 ± 8.5	35.8 ± 9.8^*^
RAVI_min_ (mL/m^2^)	18.2 ± 5.1	15.6 ± 4.9	20.0 ± 6.0	16.8 ± 5.8	19.0 ± 7.3	14.0 ± 5.0^*^	19.1 ± 6.2	15.5 ± 5.3	17.3 ± 6.0^*^
LAEF_t_ (%)	65.7 ± 8.2	69.4 ± 6.1	61.9 ± 9.8	64.4 ± 6.7	57.7 ± 9.3	60.3 ± 8.7	61.8 ± 9.6^†^	64.7 ± 8.1^†^	63.2 ± 9.0^†^
LAEF_p_ (%)	42.8 ± 10.5	48.7 ± 9.1	35.4 ± 10.1	36.1 ± 8.5	28.4 ± 8.7	29.0 ± 8.1	35.6 ± 11.3^†^	37.9 ± 11.8^†^	36.7 ± 11.6^†^
LAEF_a_ (%)	40.2 ± 9.6	39.9 ± 8.3	41.4 ± 10.7	45.1 ± 7.6	41.0 ± 9.2	43.9 ± 11.7	40.9 ± 9.7	43.0 ± 9.5	41.9 ± 9.6
RAEF_t_ (%)	53.0 ± 6.7	53.4 ± 8.0	48.5 ± 5.7	51.8 ± 8.4^*^	51.1 ± 8.1	56.1 ± 7.4	50.9 ± 7.1	53.8 ± 8.0	52.3 ± 7.7^*^^†^
RAEF_p_ (%)	29.2 ± 9.6	31.0 ± 8.4	18.8 ± 9.9	21.2 ± 9.6	19.0 ± 11.3	17.0 ± 8.7	22.3 ± 11.2^†^	23.1 ± 10.6^†^	22.7 ± 10.9^†^
RAEF_a_ (%)	33.2 ± 9.7	32.3 ± 8.3	37.1 ± 9.5	38.4 ± 9.3	39.0 ± 9.6	47.0 ± 7.7	36.5 ± 9.8	39.2 ± 10.3^†^	37.8 ± 10.1^†^

*Results are reported as mean ± SD*.

* *P < 0.05: males vs. females*.

† *P < 0.05: age-related difference among three age groups*.

*HR, heart rate; SBP, systolic blood pressure; DBP, diastolic blood pressure; LAVI_max_, maximal left atrial volume index; LAVI_min_, minimal left atrial volume index; RAVI_max_, maximal right atrial volume index; RAVI_min_, minimal right atrial volume index; LAEF_t_, left atrial total emptying fraction; LAEF_p_, left atrial passive emptying fraction; LAEF_a_, left atrial active emptying fraction; RAEF_t_, right atrial total emptying fraction; RAEF_p_, right atrial passive emptying fraction; RAEF_a_, right atrial active emptying fraction*.

### Normal Values of LA and RAFW Strains

The normal strain data of the whole population and each gender and age subgroups are presented in Table 2. RS and LS of LA were 16.7 ± 4.4% and 30.7 ± 10.2% for reservoir function, 8.1 ± 2.9% and 19.5 ± 8.2% for conduit function, and 8.7 ± 3.0% and 10.9 ± 3.7% for contractile function. The amplitudes of RAFW strains were larger than LA strains (*P* < 0.001) except for conduit RS (8.1 ± 3.9% vs. 8.1 ± 2.9%, *P* = 0.849). According to LS data, conduit function during early diastole plays a predominant role in ventricular filling in comparison with booster pump function during late diastole (LA: 19.5 ± 8.2% vs. 10.9 ± 3.7%; RAFW: 33.3 ± 14.2% vs. 19.1 ± 8.5%).

**Table 2:** Bi-atrial reservoir, conduit and pump radial, and longitudinal strains.

	**Healthy volunteers (*n* = 150)**	**Gender subgroups**		**Age subgroups**		
		**Male (*n* = 75)**	**Female (*n* = 75)**	**G_**20−40**_ (*n* = 50)**	**G_**41−60**_ (*n* = 50)**	**G_**61−80**_ (*n* = 50)**
**LA**						
LA reservoir radial strain (%)	16.7 ± 4.4	16.6 ± 4.6	16.8 ± 4.3	18.1 ± 3.6	17.0 ± 4.4	15.0 ± 4.8
LA conduit radial strain (%)	8.1 ± 2.9	7.9 ± 2.7	8.2 ± 3.1	9.7 ± 2.3	7.9 ± 2.5	6.6 ± 3.0
LA pump radial strain (%)	8.7 ± 3.0	8.8 ± 3.2	8.6 ± 2.8	8.5 ± 2.8	9.2 ± 3.2	8.4 ± 3.0
LA reservoir longitudinal strain (%)	30.7 ± 10.2	29.0 ± 9.9	32.3 ± 10.3	33.6 ± 8.8	31.4 ± 10.1	27.1 ± 10.8
LA conduit longitudinal strain (%)	19.5 ± 8.2	18.3 ± 7.4	20.7 ± 8.8	23.7 ± 7.8	19.6 ± 7.2	15.3 ± 7.5
LA pump longitudinal strain (%)	10.9 ± 3.7	10.3 ± 3.6	11.5 ± 3.8	10.3 ± 3.5	11.1 ± 3.4	11.3 ± 4.3
**RAFW**						
RAFW reservoir radial strain (%)	20.6 ± 5.8^*^	19.2 ± 5.3	22.1 ± 6.0	20.4 ± 6.2	19.2 ± 5.0	22.3 ± 5.8
RAFW conduit radial strain (%)	8.1 ± 3.9	7.5 ± 3.8	8.8 ± 4.0	8.3 ± 4.0	7.6 ± 3.8	8.4 ± 4.0
RAFW pump radial strain (%)	12.5 ± 4.7^*^	11.7 ± 4.7	13.3 ± 4.6	12.0 ± 4.5	11.6 ± 4.1	13.9 ± 5.2
RAFW reservoir longitudinal strain (%)	52.2 ± 17.6^*^	46.1 ± 16.0	58.2 ± 17.1	58.1 ± 16.8	47.7 ± 15.5	50.8 ± 19.1
RAFW conduit longitudinal strain (%)	33.3 ± 14.2^*^	28.9 ± 13.4	37.6 ± 13.8	40.5 ± 13.9	29.3 ± 11.5	30.3 ± 14.6
RAFW pump longitudinal strain (%)	19.1 ± 8.5^*^	17.5 ± 8.4	20.7 ± 8.2	18.1 ± 7.4	18.4 ± 8.9	20.9 ± 9.0

* *P < 0.05: LA strains vs. RAFW corresponding strains among all healthy volunteers (n = 150)*.

### Gender- and Age-Related Differences of LA and RAFW Strains

**Figure 3:**
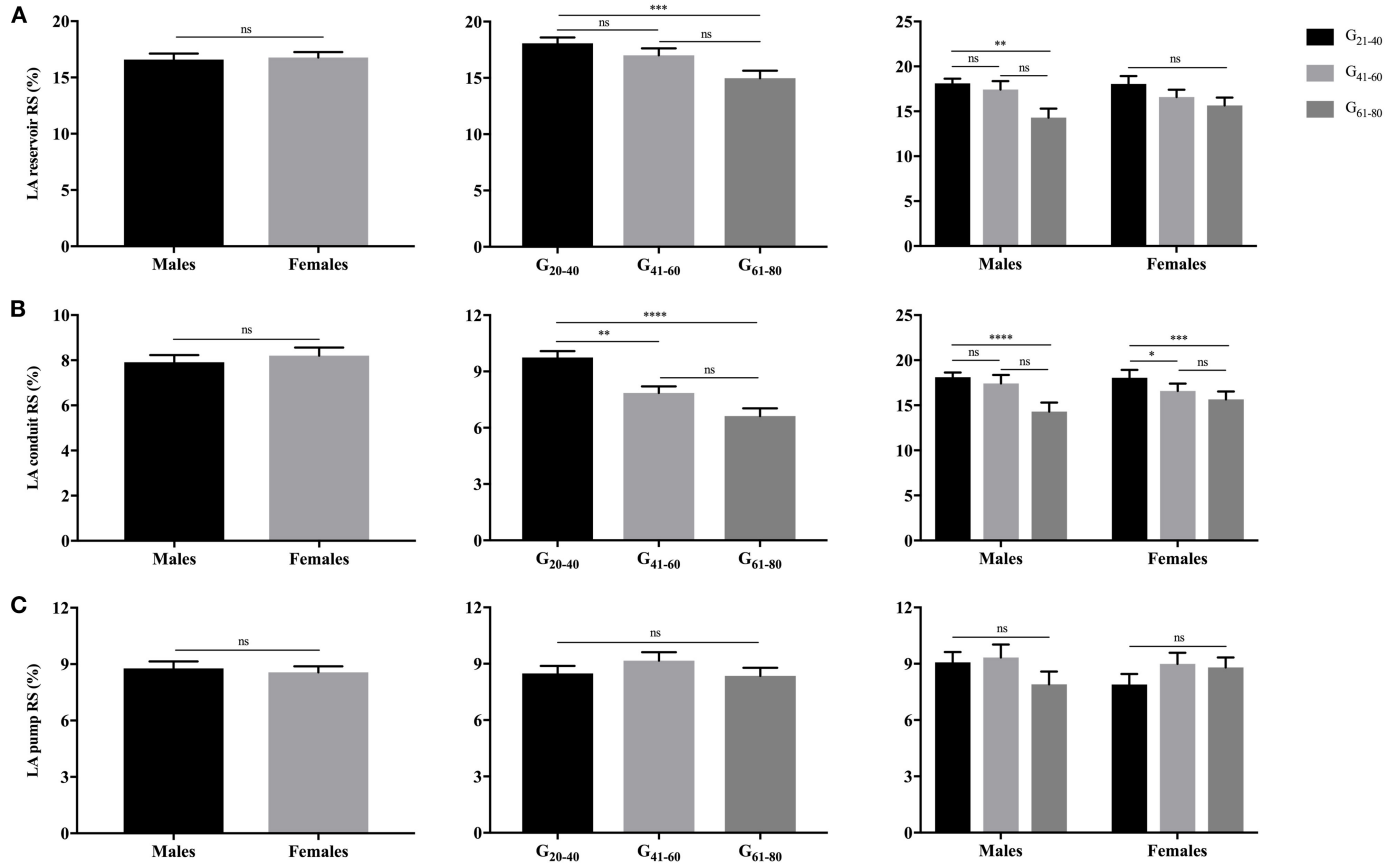
Gender- and age-related differences of LA reservoir RS **(A)**, conduit RS **(B)**, and pump RS **(C)**. *P < 0.05, **P < 0.01, ***P < 0.001, ****P < 0.0001: age-related difference; ns, not significant.

**Figure 4:**
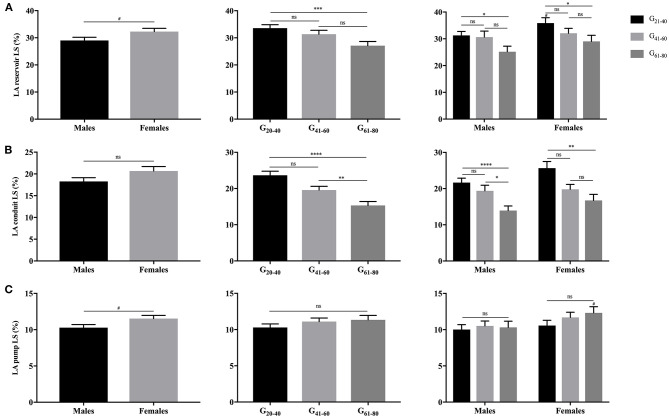
Gender- and age-related differences of LA reservoir LS **(A)**, conduit LS **(B)**, and pump LS **(C)**. ^**#**^*P* < 0.05: gender-related difference; *P < 0.05, **P < 0.01, ***P < 0.001, ****P < 0.0001: age-related difference; ns: not significant.

As illustrated in Figures 3, 4, females had significantly larger LA reservoir LS (32.3 ± 10.3% vs. 29.0 ± 9.9%, *P* = 0.029) and pump LS (11.5 ± 3.8% vs. 10.3 ± 3.6%, *P* = 0.018) than males, while no significant difference of conduit strain was detected (20.7 ± 8.8% vs. 18.3 ± 7.4%, *P* = 0.103). In regard to LARS, no gender-related differences were detected. With the increase of age, both of LA reservoir and conduit RS and LS decreased, whereas pump strains were unaffected.

**Figure 5:**
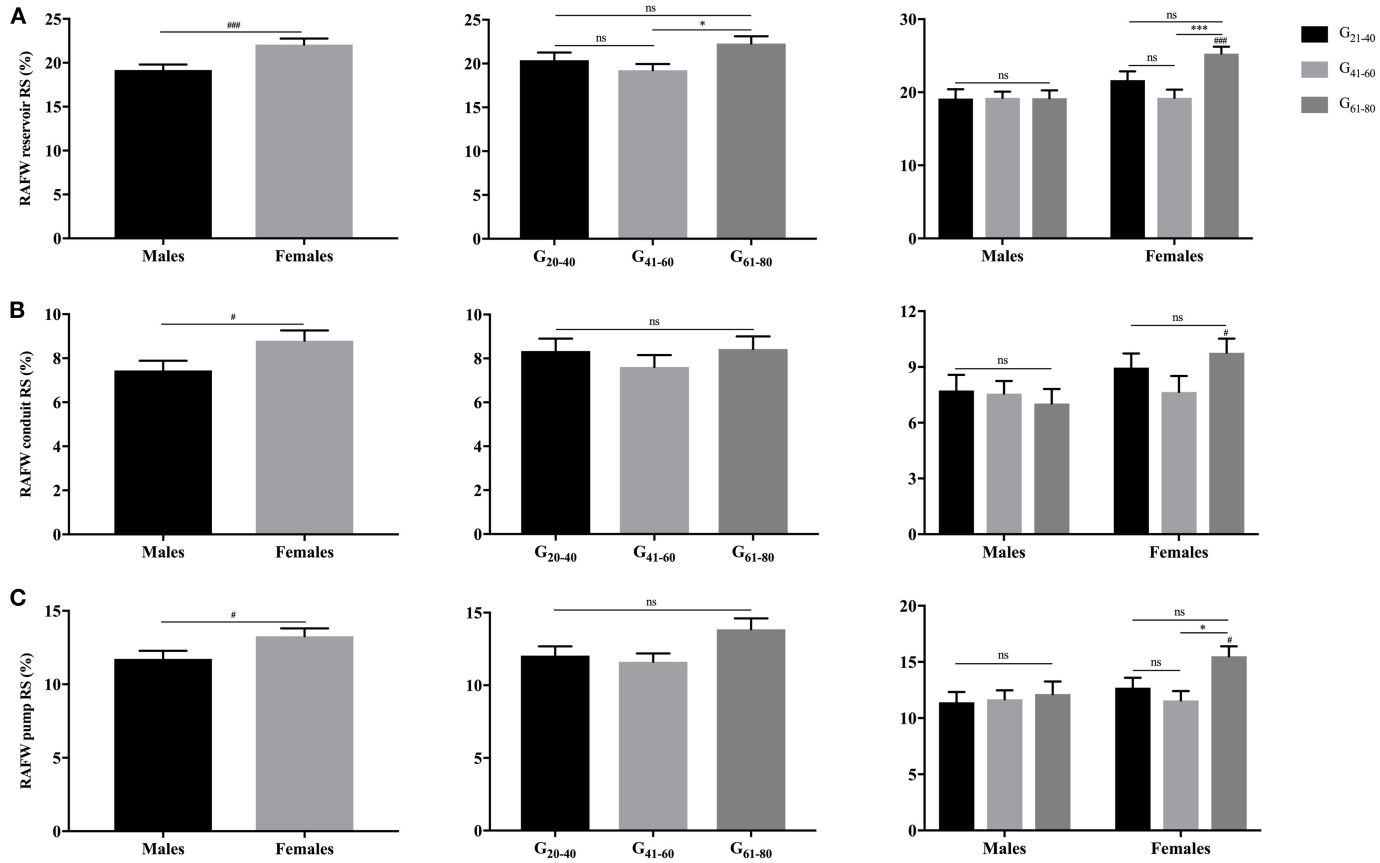
Gender- and age-related differences of RAFW reservoir RS **(A)**, conduit RS **(B)**, and pump RS **(C)**. ^**#**^*P* < 0.05, ^**###**^*P* < 0.001: gender-related difference; *P < 0.05, ***P < 0.001: age-related difference; ns, not significant.

**Figure 6:**
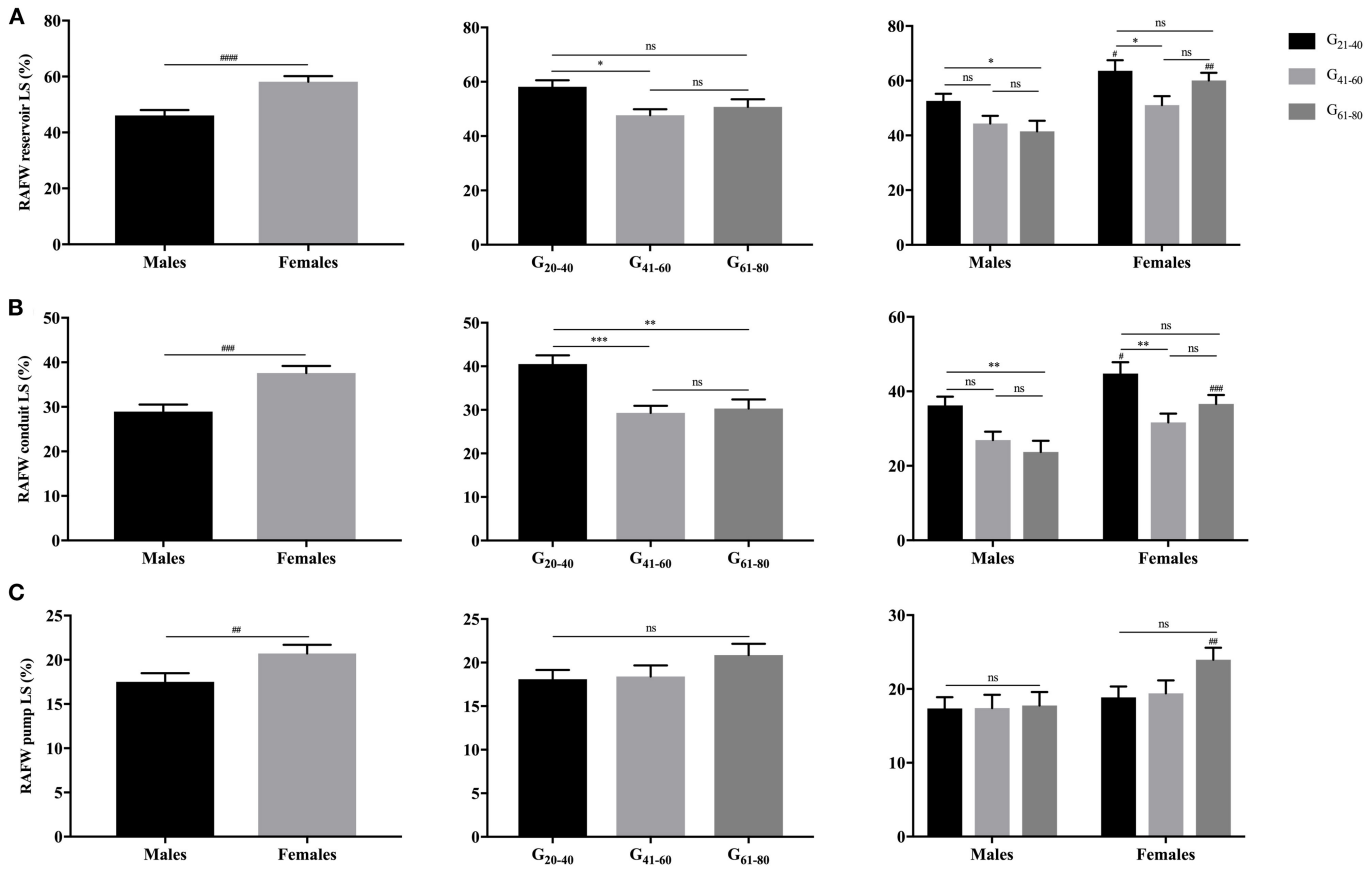
Gender- and age-related differences of RAFW reservoir LS **(A)**, conduit LS **(B)**, and pump LS **(C)**. ^##^*P* < 0.01, ^###^*P* < 0.001, ^####^*P* < 0.0001: gender-related difference; *P < 0.05, **P < 0.01, ***P < 0.001; ns, not significant.

Females showed significantly larger RAFW-RS and LS with reservoir, conduit and booster pump components than males (*P* < 0.05). While, the decrement of RAFW-LS was merely observed with reservoir and conduit function, and pump strain remained unaffected with aging (Figures 5, 6).

### Clinical Parameters Correlated With LA and RAFW Strains

A correlation between the amplitude of atrial strains and a series of clinical variables were observed (Tables 3, 4). The amplitudes of both LA and RAFW strains were negatively correlated with age (|*r*| = 0.18–0.48, *P* < 0.05), heart rate (|*r*| = 0.18–0.29, *P* < 0.05), maximal atrial volume index (|*r*| = 0.17–0.29, *P* < 0.05), minimal atrial volume index (|*r*| = 0.22–0.47, *P* < 0.05), and positively correlated with EF_t_ (*r* = 0.19–0.49, *P* < 0.05), EF_p_ (*r* = 0.19–0.45, *P* < 0.05) and EF_a_ (*r* = 0.19–0.38, *P* < 0.05). RAFW pump strains were positively correlated with RVEF (*r* = 0.33, *P* < 0.05).

**Table 3:** Correlation between left atrial strains and baseline variables.

	**Age**	**SBP**	**DBP**	**HR**	**LVEF**	**RVEF**	**LAVI_**max**_**	**LAVI_**min**_**	**LAEF_**t**_**	**LAEF_**p**_**	**LAEF_**a**_**
**LA**											
LA reservoir radial strain	−0.34^*^	−0.11	0.01	0.18^*^	0.01	−0.07	−0.29^*^	−0.47^*^	0.49^*^	0.30^*^	0.32^*^
LA conduit radial strain	−0.48^*^	−0.09	0.04	0.12	−0.06	−0.18^*^	−0.17^*^	−0.37^*^	0.44^*^	0.44^*^	0.13
LA pump radial strain	−0.04	−0.07	−0.01	0.20^*^	0.14	0.13	−0.28^*^	−0.33^*^	0.26^*^	0.01	0.35^*^
LA reservoir longitudinal strain	−0.32^*^	−0.05	−0.04	0.19^*^	0.07	0.04	−0.21^*^	−0.38^*^	0.44^*^	0.34^*^	0.19^*^
LA conduit longitudinal strain	−0.46^*^	−0.07	0.01	0.19^*^	−0.01	−0.06	−0.19^*^	−0.37^*^	0.44^*^	0.45^*^	0.06
LA pump longitudinal strain	0.09	−0.06	−0.12	0.14	0.23^*^	0.27^*^	−0.21^*^	−0.24^*^	0.19^*^	−0.01	0.33^*^

* *P < 0.05: implying significant*.

*LA, left atrial; SBP, systolic blood pressure; DBP, diastolic blood pressure; HR, heart rate; LVEF, left ventricular ejection fraction; RVEF, right ventricular ejection fraction; VI_max_, maximal volume index; VI_min_, minimal volume index, EF_t_, total emptying fraction; EF_p_, passive emptying fraction; EF_a_, active emptying fraction*.

**Table 4:** Correlation between right atrial strains and baseline variables.

	**Age**	**SBP**	**DBP**	**HR**	**LVEF**	**RVEF**	**RAVI_**max**_**	**RAVI_**min**_**	**RAEF_**t**_**	**RAEF_**p**_**	**RAEF_**a**_**
**RAFW**											
RAFW reservoir radial strain	0.13	0.01	−0.12	0.20^*^	0.16	0.27^*^	−0.16	−0.27^*^	0.25^*^	0.09	0.32^*^
RAFW conduit radial strain	−0.04	−0.14	−0.13	−0.04	−0.03	−0.01	−0.07	−0.05	0.03	0.04	0.01
RAFW pump radial strain	−0.18^*^	0.13	−0.01	0.28^*^	0.21^*^	0.33^*^	−0.17^*^	−0.28^*^	0.27^*^	0.16	0.38^*^
RAFW reservoir longitudinal strain	−0.17^*^	0.09	−0.05	0.29^*^	0.13	0.24^*^	−0.07	−0.22^*^	0.31^*^	0.19^*^	0.12
RAFW conduit longitudinal strain	−0.32^*^	−0.03	−0.04	0.21^*^	0.03	0.11	−0.01	−0.09	0.22^*^	0.32^*^	−0.04
RAFW pump longitudinal strain	0.14	0.20^*^	−0.04	0.21^*^	0.27^*^	0.33^*^	−0.16	−0.29^*^	0.31^*^	−0.04	0.33^*^

* *P < 0.05: implying significant*.

*RA, right atrial; RAFW, right atrial free wall; SBP, systolic blood pressure; DBP, diastolic blood pressure; HR, heart rate; LVEF, left ventricular ejection fraction; RVEF, right ventricular ejection fraction; VI_max_, maximal volume index; VI_min_, minimal volume index; EF_t_, total emptying fraction; EF_p_, passive emptying fraction; EF_a_, active emptying fraction*.

### Intra-observer Reproducibility

The intra-observer reproducibility was tested in 20 randomly selected individuals and resulted to be good (Table 5). As presented, CoV ranged between 10.12–12.59% and 12.62–17.04% for LARS and LALS measurement, and 15.25–27.36% and 10.80–21.39% for RAFW-RS and LS measurement, respectively. The reproducibility of LA strains appeared better than RAFW strains, and the measurement of reservoir and conduit strains was more reproducible in comparison with pump strain except for RAFW-RS.

**Table 5:** Intra-observer reproducibility of LA and RA strains.

	**MD ± SD (%)**	**ICC (95% CI)**	**CoV (%)**
**LA**			
LA reservoir radial strain	2.26 ± 1.93	0.68 (0.20–0.88)	10.12
LA conduit radial strain	1.32 ± 0.97	0.81 (0.51–0.92)	10.81
LA pump radial strain	1.26 ± 1.27	0.86 (0.64–0.94)	12.59
LA reservoir longitudinal strain	5.36 ± 4.73	0.69 (0.21–0.88)	12.62
LA conduit longitudinal strain	3.67 ± 3.08	0.76 (0.38–0.90)	13.15
LA pump longitudinal strain	2.29 ± 2.39	0.89 (0.73–0.96)	17.04
**RAFW**			
RA reservoir radial strain	3.22 ± 3.15	0.81 (0.51–0.92)	15.25
RA conduit radial strain	2.55 ± 2.20	0.74 (0.35–0.90)	27.36
RA pump radial strain	2.48 ± 2.82	0.82 (0.54–0.93)	22.33
RA reservoir longitudinal strain	6.92 ± 6.10	0.90 (0.73–0.96)	11.76
RA conduit longitudinal strain	5.05 ± 3.57	0.93 (0.82–0.97)	10.80
RA pump longitudinal strain	4.20 ± 4.03	0.86 (0.64–0.94)	21.39

*MD, mean difference; SD, standard deviation; ICC, intra-class correlation coefficient; CI, confidence interval; CoV, coefficient of variation*.

## Discussion

To the best of our knowledge, we present the first study comprehensively investigating gender- and age-associated normal values of both LA and RA phasic radial and longitudinal strains with CMR-TT modality for a rather large healthy population. In this study, we could demonstrate: (i) CMR-TT was a feasible and reproducible technique to quantify strain-based LA and RA functions; (ii) the amplitudes of the majority of atrial reservoir, conduit and pump strains were larger in females; (iii) atrial volumes along with total and passive EF decreased with aging, and go along with the following decrease of reservoir and conduit strains; (iv) the amplitudes of RAFW strains resulted to be larger than LA corresponding strains except for conduit RS; and (v) the significant associations between atrial strains and volume indices or emptying fraction implied that strain might act as a biomarker of the alteration of atrial size and function.

### Atrial Function and Strain

LA and RA play dynamic roles during separate stages of cardiac cycle to assist the LV and RV filling. The compositions of atrial function include: (i) reservoir function, storing venous blood during ventricular contraction and isovolumic relaxation; (ii) conduit function, allowing the passive blood flowing from coronary and systemic veins to ventricles in early diastole; (iii) booster pump function, accelerating ventricular filling through active contraction during late diastole. These functions can be modulated by atrial size, compliance, pre-load (venous return) along with ventricular relaxation, compliance and end-diastolic pressure (1, 21, 22).

Traditionally, atrial function was indirectly evaluated with diameter or volumetric method, which are static and unable to record the dynamic status of myocardial deformation. In contrast, strain overcomes the aforementioned disadvantages and has been reported to directly reflect atrial functions and detect atrial dysfunction in a series of disease. Moreover, atrial strain is less pre-load dependent than atrial volume index (5, 23). Unfortunately, strain has not been widely assessed in clinical routine due to the lack of reliable measurement tools and normal values.

In this study, we applied CMR-TT to quantify LA and RA strains. CMR-TT has been proven reproducible and reliable in assessing LV and RV myocardial deformation (12, 15). Recently, its application to atrial strain quantification has been gradually increasing. However, the understanding of atrial strains, especially for RA strains, is still limited. As reported in Truong et al.'s study, CMR-TT was more accurate and reproducible in measuring atrial strains compared with two-dimensional (2D) STE due to the high spatial resolution of CMR (16). Considering the deteriorated alteration of atrial functions with normal aging and differences of atrial size between males and females demonstrated by volumetric analysis (20), we analyzed LA and RA radial and longitudinal strains with reservoir, conduit and booster pump components for each individual gender and age subgroup.

### LA Strain

In this study, the normal values of LALS were similar with the data from a small sample study using CMR-TT (36.6 ± 9.3%, 23.97 ± 8.33%, and 12.63 ± 4.49%) (16). It was reported that LALS of reservoir, conduit and booster pump functions were remarkably decreased in the setting of heart failure, while conduit LS [hazard ratio (HR) = 0.68; 95% CI, 0.52–0.89; *P* = 0.006] and reservoir LS (HR = 0.66; 95% CI, 0.49–0.88; *P* = 0.004) rather than pump LS were independently predictive biomarkers of the risk of incident death or hospitalized heart failure (19).

Because of the normal aging, a series of physiological alterations including the increase of blood pressure, myocardial fibrous content and the related increased stiffness as well as the decrease of LV diastolic function and relaxation occurred. Our results demonstrated a significant decrement of reservoir and conduit LARS and LALS among age groups, whereas the pump strains remained unchanged. Both reservoir and conduit strains were negatively correlated with LAVI_max_ and LAVI_min_, and positively with LAEF_t_ or LAEF_p_. We hypothesize that the reduction is related with atrial enlargement and stiffness with aging. It's a consensus that LA conduit function declines and booster function increases with aging to promote LV filling (24). Even though LA pump strain was correlated with LAEF_a_, it remained unaffected. With regard to the effect of aging on LA pump strain controversial studies have been reported. Sun et al. reported an increase in atrial pump strain with aging (25), whereas Meel et al. didn't detect significant change in this parameter (24).

The gender-related differences were merely observed with several LA strain indices in the present study. Females showed significantly larger amplitudes of reservoir and pump LALS along with an increased tendency of conduit LALS compared with males. Sanchis et al. also reported that females had larger LA strain in comparison with males (35.37 ± 9.58% and 31.72 ± 8.15%, *P* < 0.05), however, strain values corresponding with each phasic function were not addressed in detail (26).

### RA Strain

In comparison with the number of studies investigating LA morphology, function and mechanics, few investigators characterized RA parameters. Considering the feasibility and convenience to generate both of LA and RA strain data simultaneously in the clinical practice, strains were exclusively assessed along RAFW rather than the complete myocardium. Leng et al. obtained reservoir, conduit, and pump RALS as 53.9 ± 7.8%, 33.7 ± 8.2%, and 20.2 ± 5.6% in 80 healthy participants (27), which were similar with ours. The reservoir, conduit and pump RALS rather than RAFW-LS were reported as 35.53 ± 14.35%, 22.57 ± 11.08%, and 12.96 ± 6.36% in Truong et al.'s study using CMR-TT (16), and 44 ± 10%, 27 ± 9%, 17 ± 4% in Peluso et al.'s study using 2D STE (22), respectively. Differences in analysis software and technique as well as enrolled subjects do not allow for a direct comparison of the derived values. However, the revealed predominant role of conduit strain is in good concordance with studies mentioned above.

Females showed significantly larger amplitudes of reservoir, conduit and pump RAFW-RS and LS, especially in the elderly (61–80 years of age). Our results are partly consistent with prior studies in which gender-related differences were merely observed with reservoir and conduit LS (16, 22). As revealed in the correlation analysis, RA strain was positively correlated with RVEF and RAEF, and negatively correlated with RAVI_min_ at a weak degree. Females had smaller RA size and larger RVEF (65.5 ± 6.6% vs. 60.8 ± 6.9%, *P* < 0.05) (12) and RAEF (Table 1), which facilitated the myocardial deformation and hence derived larger strain values. In general reservoir and conduit RAFW-LS decreased with normal aging, while the reservoir and pump RAFW-RS of females increased between G_41−60_ and G_61−80_. Since there were no other studies investigating RARS, it's difficult to interpret such phenomenon. We hypothesized that the discrepancy may be related with the different reproducibility between LS and RS, or maybe the increased RS is a compensation of decreased longitudinal deformation.

Of note, our study also provided some insight into the relationship between LA and RAFW strains. We demonstrated that the majority of RAFW strains were significantly larger than the corresponding LA strains, expect for conduit RS.

### Intra-observer Reproducibility of Atrial Strains

The intra-observer CoV of atrial strain assessment ranged from 10.12 to 27.36%, which was obviously larger than that of LV global strains (0.57–6.31%) and RVFW-LS (3.01%) evaluation (12), which may be attributed to the still initial stage of the analysis software and more complex morphology of atrium. A superior intra-observer reproducibility of RAFW-LS than of RAFW-RS (CoV = 10.80–21.39% vs. 15.25–27.36%) and of atrial reservoir and conduit LS than pump LS (CoV = 10.80–13.15% vs. 17.04–21.39%) was observed, which was in agreement with prior studies using CMR-TT (16) and STE (22). These findings imply a potential role of CMR-TT for atrial strain quantification, especially for reservoir and conduit LS.

## Limitations

There were some limitations with the current study. Firstly, this study was performed in a single center, and a larger multi-center study is needed for establishing more robust normal values. Secondly, we focused on RAFW rather than the whole RA myocardium given the potential influence of LA, hence the values may not directly be usable in all cases. Thirdly, comparison between CMR-TT and other modalities (e.g., STE and tissue tagging) was not performed. However, a previous study has confirmed the superior reproducibility of CMR-TT compared to 2D-STE (16) and proved the good intra-observer reproducibility of the former. Last but not least, we didn't investigate the diagnostic and prognostic role of atrial strains in diseased conditions here, further studies may be performed in the future.

## Conclusion

Our study suggests quantification of LA and RA radial and longitudinal strains with reservoir, conduit and booster pump components using CMR-TT, which is a simple, feasible and reproducible modality for strain quantification. Age- and gender-related differences of atrial volumetric indices were identified and the gender- and age-specific normal values of bi-atrial strains were established among 150 healthy volunteers. However, a population-based study is still required for establishing a more robust reference range.

## Data Availability Statement

The original contributions presented in the study are not included in the article/supplementary material, further inquiries can be directed to the corresponding author.

## Ethics Statement

The studies involving human participants were reviewed and approved by Ethics Committee of Ulm University Medical Center. The patients/participants provided their written informed consent to participate in this study.

## Author Contributions

VR was responsible for the design and overall investigation. Y-YQ was responsible for the measurement, data collection, statistical analysis, and manuscript. DB completed the recruitment of eligible participants. VR, Y-YQ, DB, and G-SM have made substantial contributions to analysis and interpretation of data, drafting, or revising the manuscript. All authors read and approved the final manuscript.

## Conflict of Interest

The authors declare that the research was conducted in the absence of any commercial or financial relationships that could be construed as a potential conflict of interest.

## Abbreviations

ANOVA: Analysis of variance
CI: Confidence interval
CMR-TT: Cardiovascular magnetic resonance based tissue tracking
CoV: Coefficent of variation
EF: Ejection fraction/Emptying fraction
EF_a_: Active emptying fraction
EF_p_: Passive emptying fraction
EF_t_: Total emptying fraction
ICC: Intra-class correlation coefficient
LA: Left atrium/Left atrial
LS: Longitudinal strain
LV: Left ventricle/Left ventricular
*r*: Correlation coefficient
RA: Right atrium/Right atrial
RAFW: Right atrial free wall
RS: Radial strain
RV: Right ventricle/Right ventricular
STE: Speckle tracking echocardiography
V_max_: Maximal volume
V_min_: Minimal volume
VI_max_: Maximal volume index
VI_min_: Minimal volume index
V_preA_: Pre-atrial contraction volume
2D: Two-dimensional.
